# Tetramethylpyrazine Confers Protection Against Oxidative Stress and NLRP3-Dependent Pyroptosis in Rats with Endometriosis

**DOI:** 10.1080/15476278.2025.2460261

**Published:** 2025-02-18

**Authors:** Ke Xu, Mingzhe Zhang, Xiaofeng Zou, Mingyang Wang

**Affiliations:** Department of Gynecology, Affiliated Hospital of Zunyi Medical University, Zunyi, Guizhou, China

**Keywords:** Endometriosis, NLRP3 inflammasome, Nrf2/HO-1 pathway, oxidative stress, tetramethylpyrazine

## Abstract

Tetramethylpyrazine (TMP) has been confirmed to suppress inflammation in endometriosis (EMs). Herein, this study investigated whether and how TMP affected NLRP3 inflammasomes and oxidative stress in EMs. After establishment of an EMs rat model, rats were treated with different concentrations of TMP. The size of endometriotic lesions and the latency and frequency of torsion in rats were recorded, followed by the measurement of relevant indicators (TNF-α, IL-6, IL-2, IL-10, MDA, SOD, GSH, CAT, ROS, NLRP3, ASC, GSDMD, caspase-1, Nrf2, and HO-1). The study experimentally determined that TMP treatment markedly decreased the size of endometriotic lesions and improved torsion in rats with EMs. The levels of inflammatory proteins, oxidative stress markers, NLRP3 inflammasome, and pyroptotic proteins were elevated in rats with EMs, all of which were reversed upon TMP treatment. Additionally, the activities of SOD, GSH, and CAT were lowered in rats with EMs, which were partly abrogated by TMP treatment. Furthermore, the downregulation of Nrf2 and HO-1 was counteracted by TMP treatment. To sum up, TMP represses excessive oxidative stress, NLRP3 inflammasome activation, and pyroptosis in rats with EMs. Additionally, TMP may activate the Nrf2/HO-1 pathway.

## Introduction

Endometriosis (EMs) is a chronic inflammatory gynecological disorder encountered by around 10% of females, which is featured by extra-uterine development of endometrial tissues and can cause debilitating painful symptoms and infertility in many patients.^1,2^ The onset of EMs is tightly correlated with retrograde menstruation, persistent ovulation, and release of cycling steroid hormones.^3^ Estrogen-dependency and progesterone-resistance are two vital events during the progression of EMs, which result in oxidative stress and inflammation.^4^ As a pivotal process in inflammation, the activation and formation of NLRP3 inflammasomes is a critical modulator in pyroptosis that assumes a role in the pathophysiology of EMs.^5^ These findings suggest the involvement of NLRP3-regulated pyroptosis in the disease progression. Accordingly, research on oxidative stress and NLRP3-dependent pyroptosis in EMs contributes to a deeper understanding of disease pathogenesis and the development of therapies for EMs. Notably, it is urgently needed to seek new treatments and drugs for EMs since current therapies for EMs have limited efficacy, with obvious side effects and risks and high symptom recurrence rates.^6^

Tetramethylpyrazine (TMP), also named ligustrazine, is a principal active ingredient of traditional Chinese medicine Ligusticum Chuanxiong Hort and was first isolated in 1957, which has been applied to the clinical treatment of stenocardia, coronary heart disease, and cerebral thrombosis in recent years, showing excellent therapeutic effects.^7,8^ As reported, TMP possesses a variety of physiological functions, encompassing anti-oxidative, anti-inflammation, anti-apoptotic, and neuroprotective functions.^9^ Importantly, a prior study revealed that TMP treatment led to significant reductions in lesion weight and lesional fibrosis and improvement of hyperalgesia, thus relieving EMs in mice.^10^ Another study further unraveled an anti-inflammatory property of TMP in EMs.^11^ Additionally, TMP was also found to repress oxidative stress and inflammation in mice with gestational diabetes mellitus.^12^ Likewise, TMP could impede the pyroptosis of alveolar macrophages to alleviate acute lung injury.^13^ Suppression of NLRP3 in renal tissues was demonstrated to participate in the protective role of TMP against acute kidney injury.^14^ More importantly, Li et al. observed the activation of the nuclear factor erythroid 2-related factor 2 (Nrf2)/heme oxygenase-1 (HO-1) pathway upon TMP pretreatment in the setting of lipopolysaccharide (LPS)-evoked acute lung injury.^15^ Nrf2/HO-1, an essential pathway in oxidative stress, exerts anti-inflammatory and antioxidant properties.^16^ Moreover, Nrf2/HO-1 pathway activation was indicated to engage in the alleviatory effects of methyl ester of 2-cyano-3,12-dioxooleana-1,9-dien-28-oic acid on EMs.^17^

The aforesaid findings led us to hypothesize that TMP might affect the progression of EMs by regulating NLRP3-mediated pyroptosis and the Nrf2/HO-1 pathway-associated oxidative stress. To demonstrate this hypothesis, this study assessed the impacts of TMP on oxidative stress and NLRP3-mediated pyroptosis in the rat model of EMs and discussed the potential participation of the Nrf2/HO-1 pathway in these impacts.

## Materials and methods

### Establishment of a rat model of EMs and interventions

Clean-grade adult female Sprague-Dawley rats (weight: 180–220 g; Hunan SJA Laboratory Animal Co., Ltd., Hunan, China; SCXK [Xiang] 2021–0002) were housed in cages (4 − 5 rats per cage) at 21 ± 4°C with a standardized photoperiod (lights on 07:00, off 19:00) and ad lib access to food and water. A rat model of EMs was established using the method reported in the literature.^18^ The experimentation was ratified by the Institutional Animal Care and Use Committee of Affiliated Hospital of Zunyi Medical University.

Based on the clinical dose (about 3 mg/kg Ligustrazine Phosphate Tablets for a 70 kg adult), the equivalent dose of Ligustrazine Phosphate Tablets for rats was converted by the ratio of body surface area of human and rat (the dose of rats was approximately 6.3 times that of adults)^19^ and was about 19 mg/kg. Doses for the TMP-low dose (TMP-L), TMP-medium dose (TMP-M), and TMP-high dose (TMP-H) groups were 0.5 times (8 mg/kg), 1 time (19 mg/kg), and 2 times (38 mg/kg) the equivalent dose for rats, respectively.

According to the rat model of EMs reported in the previous literature,^19^ rats were injected with estradiol valerate (1 mg/kg) 3 days before modeling to synchronize their estrous cycle. After isoflurane inhalation anesthesia and standard iodine disinfection, the uterus was exposed through a 2.5 cm midline abdominal incision. After the left horn of the uterus was ligated, the organs were preserved in normal saline solutions. The uterine horns were incised longitudinally and sutured diagonally to the internal right abdominal wall with 4–0 absorbable thread. In the sham group, the left uterine horn was excised, and the abdomen was sutured. To prevent the development of infection, gentamicin (0.1 mL) was injected intraperitoneally 3 days after surgery. For all rats, gavage was started on the first postoperative day.

All rats were randomly allocated into the following groups (5 rats per group): sham group (gavage of sham-operated rats with an equal amount of normal saline for 4 weeks), EMs group (gavage of EMs rats with an equal amount of normal saline for 4 weeks), TMP-L (gavage of EMs rats with TMP [9.5 mg/kg/d] for 4 weeks), TMP-M (gavage of EMs rats with TMP [19 mg/kg/d] for 4 weeks), TMP-H (gavage of EMs rats with TMP [38 mg/kg/d] for 4 weeks), GTN group (gavage of EMs rats with GTN [0.25 mg/kg; positive control; twice a week]),^19^ vitamin C (Vit C) group (gavage of EMs rats with Vit C [0.2 mg/kg/d; A4403; Merck]) for 4 weeks),^20^ and Indomethacin group (gavage of EMs rats with indomethacin [2.5 mg/kg; HY-14397; MedChemExpress] for 4 weeks).^21^

After the last administration, rats in the sham group were intraperitoneally injected with an equal amount of normal saline, and rats in the remaining groups were injected intraperitoneally with 50 U/kg oxytocin (HY-17571; MedChemExpress) to elicit immediate writhing responses, primarily including abdominal wall contractions, pelvic rotation, and hind limb stretches.^22^ The volume (V) of the endometrial allografts was measured with vernier calipers, and the length, width, and height of the ectopic tissue measured by vernier calipers were multiplied by 0.52 to obtain the graft volume.^23^

At the end of the experiment, rats were anesthetized with an appropriate amount of pentobarbital sodium (80 mg/kg; P3761; Sigma Aldrich, USA) and dissected. Blood was obtained from the abdominal aorta and centrifuged, and then the supernatant was harvested and stored at −80°C for later use. Afterward, rats were euthanized, and the size and shape of the ectopic foci in rats were observed. Later, surgery was performed to remove the uterus and the ectopic foci. Some of the tissues were placed in 4% paraformaldehyde solutions, while the remaining tissues were placed in liquid nitrogen for immediate freezing and stored at −80°C for subsequent experiments.

### Hematoxylin-eosin (HE) staining

Ectopic endometrial tissues of all rats were obtained, fixed in 4% formaldehyde, routinely dehydrated (1 min/time), cleared with xylene two times, and cooled on the cold table of a paraffin embedding station. Paraffin-embedded sections were deparaffinized and hydrated, followed by 10-min staining with hematoxylin (PT001, Bogoo, Shanghai, China) at ambient temperature, 30-s differentiation with 1% hydrochloric alcohol, and 1-min staining with eosin (0001-H, Xinhua LvYuan Science and Technology Co., Ltd., Beijing, China) at ambient temperature. The sections were subjected to conventional procedures. The sections were mounted with neutral balsam in a fume hood and finally photographed under a Zeiss fluorescence microscope (PrimoStar iLED, Bioresearch, Beijing, China) for morphological observation of the ectopic endometrial tissues.

### Enzyme-linked immunosorbent assay (ELISA)

The concentrations of tumor necrosis factor alpha (TNF-α), interleukin (IL)-2, IL-6, IL-10, IL-1β, and IL-18 were measured as instructed in the manuals of corresponding ELISA kits (Abcam, Cambridge, UK; ab236712, ab221834, ab234570, ab124566, ab255730, and ab213909). In brief, the aforementioned kits were equilibrated at indoor temperature for 20 min to prepare washing solutions. To plot the standard curve, 100 μL of standard samples were dissolved and loaded onto the reaction plate, followed by 90 min of incubation at 37°C. After washing, the reaction plate was added with 100 μL biotinylated antibody solutions for a further 60-min incubation at 37°C. After that, the plate was incubated with 100 μL of fresh reaction working solutions containing enzymes at 37°C for 30 min. The plate was incubated with substrates (100 μL) for 15 min at 37°C in the shade after three rinses. Within 3 min following reaction termination, a microplate reader (BioTek Instruments Inc., Winooski, VT) was utilized for optical density detection at 450 nm.

### Detection of oxidative stress indicators

Levels of reactive oxygen species (ROS), malondialdehyde (MDA), superoxide dismutase (SOD), catalase (CAT), and glutathione (GSH) were tested with corresponding ELISA kits (Nanjing Jiancheng Bioengineering Institute, Nanjing, China). As an example, ROS levels were assayed with the ROS Assay Kit (chemiluminescence) (E004; Nanjing Jiancheng Bioengineering Institute). Briefly, rat ectopic endometrial tissues were enzymatically digested to obtain cell suspensions. The cell suspensions were cultured with 2,7-dichlorofluorescin diacetate at a concentration of 10 μM in a constant-temperature incubator at 37°C for 60 min, and the probe-labeled cell suspensions were attained for 8-min centrifugation at 1000 ×g, followed by phosphate-buffered saline (PBS) rinsing (once). The cell precipitates were harvested and resuspended with PBS. Then, the fluorescence value was determined on a spectrophotometer (exciting at 500 nm and emitting at 530 nm). Three repeated independent experiments were implemented.

### Quantitative reverse transcription-polymerase chain reaction (qRT-PCR)

The total RNA of tissues was extracted with TRIzol (15596026; Invitrogen, Carlsbad, CA) and subsequently reverse-transcribed into cDNA using the Reverse Transcription Kit (RR047A; Takara, Tokyo; Japan) with a system of 20 μL. qRT-PCR was implemented with the assistance of SYBR Premix EX Taq Kit (RR420A; Takara) in an ABI7500 PCR instrument (ABI, Foster City, CA). Reaction system consisted of 9 μL of SYBR Mix, 0.5 μL of forward primer, 0.5 μL of reverse primer, 2 μL of cDNA, and 8 μL of RNase-free H_2_O. In addition, the following reaction conditions were utilized: 12 min at 94°C and 38 consecutive cycles of 15 s at 94°C and 1 min at 58°C. Three replicate wells were set for each sample. The primer synthesis was accomplished by Sangon (Shanghai, China; Table 1). The Ct value of each well was recorded, and the relative expression of the product was calculated with the 2^−ΔΔCt^ formula. β-actin served as the internal control.

**Table 1.** Primer sequences for qRT-PCR.

Name of primer	Sequences (5’-3’)	
NRF2	F	TTGGGGTAAGTCGAGAAGTGTTT
	R	ATGTGGGCAACCTGGGAGTA
HO-1	F	AGCGAAACAAGCAGAACCCAGTC
	R	GCTGTGTGGCTGGTGTGTAAGG
β-actin	F	TGCTATGTTGCCCTAGACTTCG
	R	GTTGGCATAGAGGTCTTTACGG

F, forward; R, reverse.

### Western blotting

Tissues were added with 500 μL of Radioimmunoprecipitation Assay buffer (Pierce, Rockford, IL) containing phenylmethylsulphonyl fluoride (MA0001; MeilunBio, Dalian, China) and placed on ice for 60 min. The above-mentioned solution was cooled and transferred into a 1.5 mL Eppendorf (EP) tube before 30-min centrifugation at 4°C and 12,000 rpm. The supernatant was transferred into an EP tube, followed by the measurement of the protein concentration as instructed in the protocols of the Bicinchoninic Acid Protein Assay Kit. Afterward, 30 μg proteins in each group were adjusted to the same volume of sample loading with deionized water, and 10% separation gels and 5% concentration gels were configured for sodium dodecyl-sulfate polyacrylamide gel electrophoresis. The extracted total proteins were loaded onto each lane in equal amounts with a Finnpipette, followed by electrophoretic separation under the conditions of 0.5 h at 80 V and 1 h at 100 V. The proteins were transferred onto membranes at 15 V with the semi-dry transferring method for 10 min. Next, the membranes underwent 1 h of sealing with 5% skimmed milk-TBST and overnight incubation with primary antibodies (Abcam; antibodies were diluted as per the instructions) against β-actin (ab6272; 1:2000; the internal reference), NLRP3 (ab263899; 1:1000), apoptosis-associated speck-like protein containing a C-terminal caspase recruitment domain (ASC; ab309497; 1:1000), caspase-1 (ab286125; 1:1000), Gasdermin D N-terminal (GSDMD-N; ab239377; 1:1000), NRF2 (ab313825; 1:1000), and HO-1 (ab137749; 1:1000) at 4°C. After three TBST rinses at ambient temperature (5 min/time), goat anti-rabbit IgG secondary antibodies (HRP) (ab6721; 1:2000; Abcam) were supplemented for another 45-min incubation at 37°C, followed by three TBST rinses again. Enhanced chemiluminescence luminescent solutions were added for development, and SmartView Pro 2000 (UVCI-2100, Major Science, Saratoga, CA) was utilized for photographing. The grayscale of protein bands was analyzed with Image J software (Media Cybernetics, USA).

### Statistical analysis

All data were subjected to statistical analysis and plotting with GraphPad Prism 9.5.0 (GraphPad Software Inc., San Diego, California, USA). Measurement data were described as mean ± standard deviation. Data were compared between two groups with the *t*-test and among multiple groups with one-way analysis of variance. *p* < 0.05 denoted a statistically significant difference.

## Results

### TMP reduces the volume of ectopic lesions in rats with EMs

**Figure 1.**
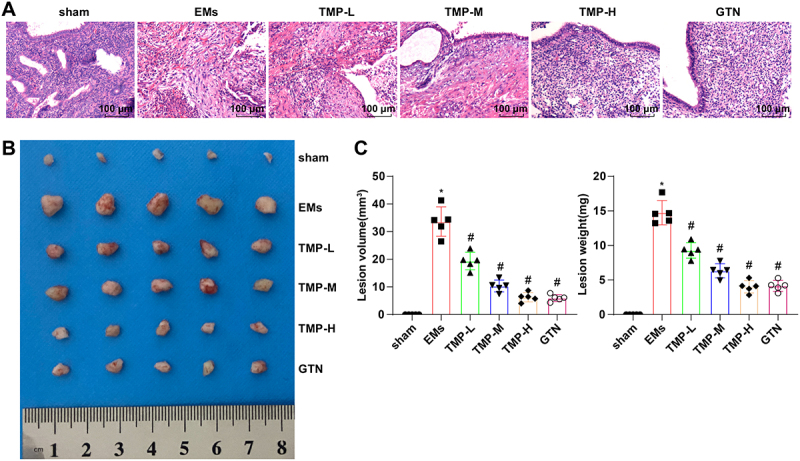
TMP reduces the size of ectopic endometrial lesions in rats with EMs.

First, the characteristics of ectopic endometrial lesions were assessed with HE staining. The results (Figure 1A) displayed that in the sham group, the endometrial epithelial cells of rats were complete and tall columnar or columnar in shape, and glandular epithelial cells were mostly columnar in shape, with neatly arranged interstitial cells and many microvilli. In the EMs group, the ectopic endometrial lesions of rats were polycystic, with the outer wall of the cysts covered with a layer of fibrous connective tissues, and low columnar endometrial epithelial cells grew in a circular or serrated pattern, with necrosis and inflammatory cell infiltration in individual areas. Additionally, the endometrial interstitial layer was thinned with fibrotic changes of various extents. Moreover, treatment with TMP or GTN improved the morphological changes of the endometrium and decreased inflammatory infiltration, and the improving effect of TMP on EMs was enhanced concentration-dependently. Meanwhile, rats in the EMs group showed torsion, with a statistically significant difference from those in the sham group (*p* < 0.05), whereas TMP or GTN treatment substantially diminished torsion in EMs rats (Table 2). Furthermore, the observation of ectopic endometrial lesions revealed that TMP treatment remarkably declined the size of the lesions in EMs rats (Figure 1B,C).

A, Pathology of the endometrium in rats observed with HE staining; B, Representative images of ectopic endometrial lesion in rats. C, Volume and weight of ectopic endometrial lesion. *p < 0.05 *vs*. the sham group, ^#^*p* < 0.05 *vs*. the EMs group. These results were measurement data described as mean ± standard deviation. Data were compared among multiple groups with one-way analysis of variance. *N* = 5.

**Table 2.** The latency and frequency of torsion in rats among groups.

Group	Number	Latency of torsion (s)	Frequency of torsion(times)
Sham	5	–	–
EMs	5	32.58 ± 9.54*	21.93 ± 7.91*
TMP(L)	5	38.24 ± 6.15^#^	15.19 ± 2.17^#^
TMP(M)	5	49.31 ± 5.34^#^	10.42 ± 1.46^#^
TMP(H)	5	53.65 ± 6.97^#^	6.05 ± 0.87^#^
GTN	5	51.43 ± 4.38^#^	7.12 ± 1.14^#^

TMP, Tetramethylpyrazine; L, low dose; M, medium dose; H, high dose; GTN, Gestrinone. *p < 0.05 *vs*. the sham group, ^#^*p* < 0.05 *vs*. the EMs group. These results were measurement data described as mean ± standard deviation. Data were compared among multiple groups with one-way analysis of variance. *N* = 5.

### TMP lowers the levels of inflammatory factors in rats with EMs

**Figure 2.**
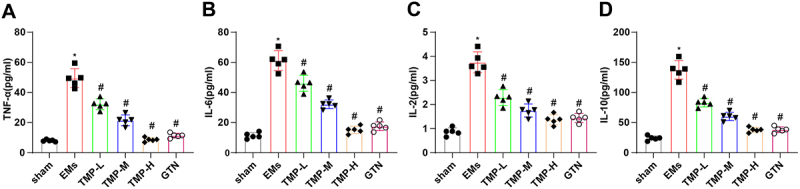
TMP decreases the levels of inflammatory factors in the serum of rats with EMs.

To analyze whether TMP could modulate inflammatory response in EMs rats, we first examined the levels of pro-inflammatory markers (TNF-α, IL-6, IL-2, and IL-10) in the serum of rats using ELISA. The results exhibited that the serum levels of the aforementioned markers were substantially higher in the EMs group versus the sham group but greatly lower in all TMP groups versus the EMs groups (Figure 2A–D). The aforesaid results suggested that TMP concentration-dependently inhibited inflammation in rats with EMs.

A-D, ELISA detection of TNF-α (A), IL-6 (B), IL-2 (C), and IL-10 (D) levels in the serum of rats. *p < 0.05 *vs*. the sham group, ^#^*p* < 0.05 *vs*. the EMs group. These results were measurement data described as mean ± standard deviation. Data were compared among multiple groups with one-way analysis of variance. *N* = 5.

### TMP alleviates excessive oxidative stress in rats with EMs

**Figure 3.**
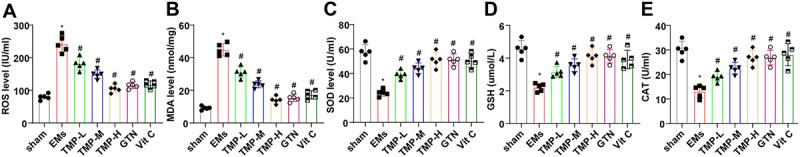
TMP exerts an anti-oxidative role in rats with EMs.

Oxidative stress is defined as an imbalance between ROS and antioxidants, which may be involved in the pathophysiology of EMs, leading to generalized inflammatory responses in the peritoneal cavity.^24^ TMP was found to have antioxidant effects.^13^ Therefore, we speculated that TMP might ameliorate excessive oxidative stress in EMs rats. The contents of ROS, MDA, SOD, GSH, and CAT, which are oxidative stress-related indicators, were measured with ELISA in rat serum. As presented in Figure 3A–E, the EMs group displayed significant elevations in contents of ROS and MDA and marked reductions in activities of SOD, GSH, and CAT as compared to the sham group, indicating that oxidative stress occurs in EMs rats. Vit C (ascorbic acid), as a strong antioxidant, has the greatest property of reducing properties.^25^ Accordingly, EMs rats were treated with Vit C. The results showed that the serum contents of ROS and MDA were obviously reduced and the activities of SOD, GSH, and CAT were remarkably enhanced in the Vit C group as compared to the EMs group (Figure 3A–E), further suggesting that oxidative stress occurred in EMs rats. Moreover, TMP treatment concentration-dependently diminished ROS and MDA contents while increasing SOD, GSH, and CAT activities in the serum of EMs rats. Overall, oxidative stress occurs in EMs rats, and TMP depressed excessive oxidative stress in EMs rats.

A-E, ELISA to determine the contents of ROS (A) and MDA (B) and the activities of SOD (C), GSH (D), and CAT (E) in the serum of rats. *p < 0.05 *vs*. the sham group, ^#^*p* < 0.05 *vs*. the EMs group. These results were measurement data described as mean ± standard deviation. Data were compared among multiple groups with one-way analysis of variance. *N* = 5.

### TMP curbs pyroptosis in rats with EMs

**Figure 4.**
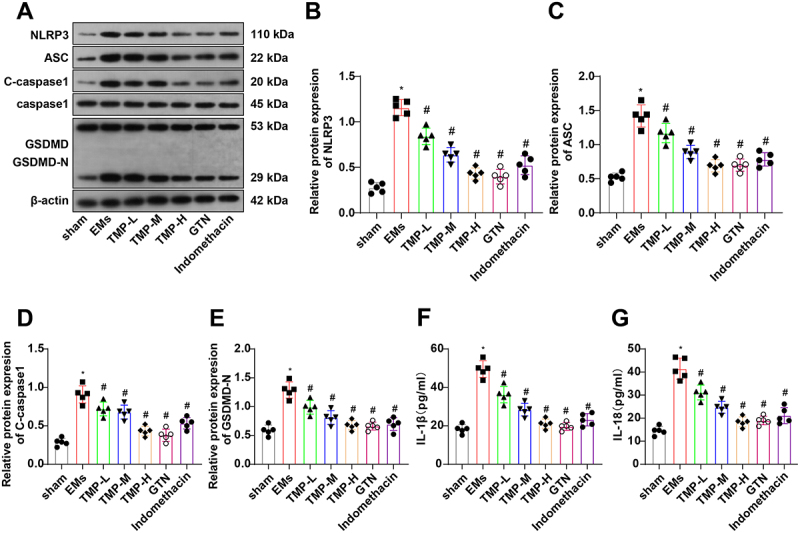
TMP exerts an anti-pyroptotic effect on rats with EMs.

The NLRP3 inflammasome pathway is implicated in the pathophysiology of EMs.^5^ Additionally, TMP was demonstrated to have anti-inflammatory effects that alleviate acute lung injury and reduce pyroptosis through the NLRP3/caspase-1 pathway.^13^ Hence, we speculated that TMP may inhibit pyroptosis in EMs. NLRP3, ASC, cleaved-caspase-1, and GSDMD-N expression was measured by western blotting, and IL-1β and IL-18 expression in rat serum was determined with ELISA. The results exhibited that NLRP3, ASC, cleaved-caspase-1, and GSDMD-N proteins were prominently upregulated (Figure 4A–E) and the serum levels of IL-1β and IL-18 were elevated (Figure 4F,G) in the EMs group versus the sham group, illustrating that the NLRP3 inflammasome pathway participated in EMs. The anti-inflammatory drug indomethacin was used to treat EMs rats. The results demonstrated a significant decrease in the expression of NLRP3, ASC, cleaved-caspase-1, and GSDMD-N (Figure 4A–E) and the serum levels of IL-1β and IL-18 in the Indomethacin group when compared to the EMs group (Figure 4F,G). TMP or GTN treatment reduced the expression of NLRP3, ASC, cleaved-caspase-1, and GSDMD-N and the serum levels of IL-1β and IL-18 in EMs rats (Figure 4A–G). Altogether, TMP treatment repressed NLRP3 inflammasome activation and pyroptosis in EMs rats.

A, Protein bands of NLRP3, ASC, cleaved-caspase-1, and GSDMD-N; B-E, NLRP3 (B), ASC (C), cleaved-caspase-1 (D), and GSDMD-N (E) expression in rats tested with western blotting; F-G, IL-1β (F) and IL-18 (G) levels in the serum of rats detected by ELISA. *p < 0.05 *vs*. the sham group, ^#^*p* < 0.05 *vs*. the EMs group. These results were measurement data described as mean ± standard deviation. Data were compared among multiple groups with one-way analysis of variance. *N* = 5.

### TMP may activate the Nrf2/HO-1 pathway to mitigate EMs in rats

**Figure 5.**
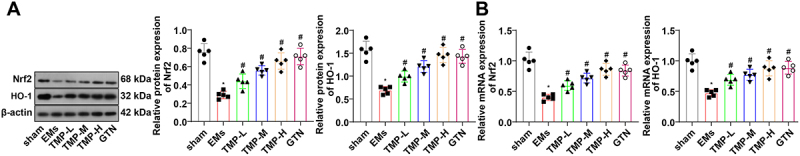
TMP may activate the Nrf2/HO-1 pathway in rats with EMs.

It has been revealed that the Nrf2/HO-1 pathway and its metabolites play critical roles in maintaining cellular redox homeostasis and in repressing pyroptosis.^26,27^ Therefore, we speculated that TMP might mediate the Nrf2/HO-1 pathway in EMs. Nrf2 and HO-1 expression in the ectopic foci of EMs rats was examined with the use of western blotting and qRT-PCR. The data demonstrated that Nrf2 and HO-1 expression in ectopic foci was decreased in EMs rats, which was concentration-dependently increased following TMP treatment (Figure 5A,B). The aforementioned results illustrated that TMP might have a therapeutic effect on EMs by activating the Nrf2/HO-1 pathway and upregulating antioxidant proteins such as HO-1.

A-B, Nrf2 and HO-1 protein (A) and mRNA (B) levels in ectopic lesions of rats determined with western blotting and qRT-PCR, respectively. *p < 0.05 *vs*. the sham group, ^#^*p* < 0.05 *vs*. the EMs group. These results were measurement data described as mean ± standard deviation. Data were compared among multiple groups with one-way analysis of variance. *N* = 5.

## Discussion

EMs can give rise to pelvic organ dysfunction, severe pelvic pain, infertility, and secondary psychiatric problems, thus dramatically compromising the health of patients.^28^ To date, EMs remains incurable, and current therapies usually are ineffective, which mainly focus on the management of pain or infertility.^29^ This challenge calls for extensive research concerning the pathological mechanisms of this disease with the hope of developing novel and effective therapies. Furthermore, TMP was observed to impede epithelial-mesenchymal transformation and then ameliorate allograft EMs when combined with ferulic acid and tetrahydropalmatine.^30^ Nevertheless, relevant studies have not yet discussed the exact mechanism of TMP in EMs. Accordingly, this study analyzed the mechanism of TMP in oxidative stress and NLRP3-dependent pyroptosis during the progression of EMs, and our experimental results elucidated that TMP hampered oxidative stress and NLRP3-regulated pyroptosis and might activate the Nrf2/HO-1 pathway in rats with EMs.

Oxidative stress is manifested with abundant ROS and deficient antioxidant mechanisms because of an imbalance between ROS and antioxidants (SOD, CAT, and GSH), which is crucial in the pathophysiology underlying EMs and also plays a role in endometriotic pain.^31,32^ In our study, ELISA results revealed that the contents of ROS and MDA increased and the activities of SOD, CAT, and GSH decreased in rats with EMs, further confirming the implication of oxidative stress in the progression of EMs. Despite its unclear effects on oxidative stress in EMs, TMP has been elucidated to depress oxidative stress in multiple diseases. For instance, a prior study clarified that TMP augmented SOD activities and diminished MDA contents to protect against oxidative stress in rats with arthritis.^33^ Li et al. noted that TMP exerted inhibitory impacts on oxidative stress, therefore preventing nitroglycerin-induced tolerance.^34^ Another study also showed that TMP treatment caused the downregulation of oxidative markers in the hippocampus of type 2 diabetic rats.^35^ Consistently, our results exhibited reductions in ROS and MDA contents and increases in SOD, CAT, and GSH activities in rats with EMs after TMP treatment, supporting the notion that TMP also suppressed oxidative stress in EMs.

It has been extensively recognized that oxidative stress can result in chronic inflammation to induce numerous chronic diseases.^36^ Also, oxidative stress and inflammation are two major pathophysiological mechanisms in EMs.^37^ Moreover, a previous study unraveled that multiple inflammatory factors, including TNF-α, IL-6, IL-2, and IL-10, were upregulated in plasma from patients with EMs.^38^ In the present study, similar trends were observed in a rat model of EMs. Furthermore, our findings elucidated that TMP treatment hindered inflammation in EMs rats by downregulating TNF-α, IL-6, IL-2, and IL-10 levels. Consistently, a recent study demonstrated that TMP treatment lowered TNF-α, IL-6, and IL-1β levels in human endometrial stromal cells from patients with EMs.^11^

NLRP3 inflammasome has been recently reported as an essential regulator of pathological inflammation in multiple diseases.^39^ NLRP3 inflammasome activation induces caspase 1-dependent production of pro-inflammatory IL-1β and IL-18 and GSDMD-regulated pyroptosis, which is a type of programmed cell death in response to inflammation.^40,41^ Additionally, activated NLRP3 inflammasomes can accelerate pyroptosis by recruiting ASC.^42^ Of note, repression of NLRP3 inflammasome-dependent pyroptosis confers protection against the development of EMs.^43^ In our study, the levels of NLRP3, ASC, cleaved-caspase-1, GSDMD-N, IL-1β, and IL-18 were detected to be higher in EMs rats than in control rats, further validating the critical role of NLRP3-associated pyroptosis in the progression of EMs. More importantly, it was observed in our study that TMP treatment diminished the levels of aforesaid markers in EMs rats, illustrating the repressive effects of TMP on NLRP3-mediated pyroptosis in EMs. Consistently, a recent study demonstrated that TMP could curtail the pyroptosis of alveolar macrophages to relieve acute lung injury.^13^ Meanwhile, TMP treatment also could ameliorate cyclophosphamide-caused hepatotoxicity by attenuating NLRP3 inflammasome-regulated pyroptosis.^44^ Altogether, these data allowed us to conclude that TMP mitigates EMs through the repression of oxidative stress, inflammation, and NLRP3-associated pyroptosis. Subsequently, the mechanism of TMP in EMs was further ascertained in the present study.

TMP has been revealed to activate the Nrf2/HO-1 pathway in several diseases. For example, TMP has suppressive effects on oxidative stress in rats with nitroglycerin-induced tolerance by upregulating Nrf2 and HO-1.^34^ TMP attenuates pro-inflammatory responses by elevating Nrf2 and HO-1 expression, thus conferring neuroprotection against cerebral ischemia.^45^ TMP treatment greatly lowered intracellular ROS levels and MDA expression while augmenting SOD expression by activating the Nrf2/HO-1 pathway in H_2_O_2_-exposed human umbilical cord mesenchymal stem cells.^46^ Such impact of TMP on the Nrf2/HO-1 pathway was also found in EMs in our study, as evidenced by the upregulation of Nrf2 and HO-1 in rats with EMs after TMP treatment. The Nrf2/HO-1 pathway exerts functions in suppressing inflammation, oxidation stress, and cell death.^47^ Of note, the Nrf2/HO-1 pathway activation depresses NLRP3 inflammasome-dependent pyroptosis in various diseases, including periodontitis, acute lung injury, and cerebral ischemia-reperfusion damage.^48–50^ In addition, another study unveiled that upregulated Nrf2 and HO-1 were involved in the antioxidant and antiapoptotic effects of Boswellia serrata gum resin extract in rats with EMs.^51^ Therefore, the mitigating effect of TMP on EMs might be achieved by activating the Nrf2/HO-1 pathway.

Conclusively, our study described the ameliorating impact of TMP on the rats with EMs and a relevant mechanism that TMP treatment protected against oxidative stress and NLRP3-dependent pyroptosis and might activate the Nrf2/HO-1 pathway in EMs. Additionally, the results also exhibited that TMP treatment repressed torsion and lesion size in the rat model of EMs. Accordingly, our findings implicate that TMP can be an excellent candidate drug for EMs treatment. However, we only examined the expression of Nrf2 and HO1 and did not perform an in-depth study and validation of this pathway in EMs. Therefore, future studies should further evaluate the impacts of the Nrf2/HO-1 pathway on EMs progression.

## Data Availability

All data generated or analyzed during this study are included in this article. Further enquiries can be directed to the corresponding author.

## References

[1] Horne AW, Missmer SA. Pathophysiology, diagnosis, and management of endometriosis. BMJ. 2022;379:e070750. doi:10.1136/bmj-2022-070750. 36375827

[2] Koninckx PR, Fernandes R, Ussia A, Schindler L, Wattiez A, Al-Suwaidi S, Amro B, Al-Maamari B, Hakim Z, Tahlak M, et al. Pathogenesis based diagnosis and treatment of endometriosis. Front Endocrinol (Lausanne). 2021;12:745548. doi:10.3389/fendo.2021.745548. 34899597 PMC8656967

[3] Bulun SE, Yilmaz BD, Sison C, Miyazaki K, Bernardi L, Liu S, Kohlmeier A, Yin P, Milad M, Wei J, et al. Endometriosis. Endocr Rev. 2019;40(4):1048–11. doi:10.1210/er.2018-00242. 30994890 PMC6693056

[4] Vannuccini S, Clemenza S, Rossi M, Petraglia F. Hormonal treatments for endometriosis: the endocrine background. Rev Endocr Metab Disord. 2022;23(3):333–355. doi:10.1007/s11154-021-09666-w. 34405378 PMC9156507

[5] Irandoost E, Najibi S, Talebbeigi S, Nassiri S. Focus on the role of NLRP3 inflammasome in the pathology of endometriosis: a review on molecular mechanisms and possible medical applications. Naunyn Schmiedebergs Arch Pharmacol. 2023;396(4):621–631. doi:10.1007/s00210-022-02365-6. 36542122

[6] Saunders PTK, Horne AW. Endometriosis: etiology, pathobiology, and therapeutic prospects. Cell. 2021;184(11):2807–2824. doi:10.1016/j.cell.2021.04.041. 34048704

[7] Zou J, Gao P, Hao X, Xu H, Zhan P, Liu X. Recent progress in the structural modification and pharmacological activities of ligustrazine derivatives. Eur J Med Chem. 2018;147:150–162. doi:10.1016/j.ejmech.2018.01.097. 29432947

[8] Shao H, He X, Zhang L, Du S, Yi X, Cui X, Liu X, Huang S, Tong R. Efficacy of ligustrazine injection as adjunctive therapy in treating acute cerebral infarction: a systematic review and meta-analysis. Front Pharmacol. 2021;12:761722. doi:10.3389/fphar.2021.761722. 34880757 PMC8646035

[9] Lin J, Wang Q, Zhou S, Xu S, Yao K. Tetramethylpyrazine: a review on its mechanisms and functions. Biomed Pharmacother. 2022;150:113005. doi:10.1016/j.biopha.2022.113005. 35483189

[10] Huang S, Xiao F, Guo SW, Zhang T. Tetramethylpyrazine retards the progression and fibrogenesis of endometriosis. Reprod Sci. 2022;29(4):1170–1187. doi:10.1007/s43032-021-00813-x. 35099777 PMC8907108

[11] Feng Y, Dong H, Zheng L. Ligustrazine inhibits inflammatory response of human endometrial stromal cells through the STAT3/IGF2BP1/RELA axis. Pharm Biol. 2023;61(1):666–673. doi:10.1080/13880209.2023.2195883. 37095705 PMC10132247

[12] Jiao Y, Zhang S, Zhang J, Du J. Tetramethylpyrazine attenuates placental oxidative stress, inflammatory responses and endoplasmic reticulum stress in a mouse model of gestational diabetes mellitus. Arch Pharm Res. 2019;42(12):1092–1100. doi:10.1007/s12272-019-01197-y. 31797253

[13] Jiang R, Xu J, Zhang Y, Zhu X, Liu J, Tan Y. Ligustrazine alleviate acute lung injury through suppressing pyroptosis and apoptosis of alveolar macrophages. Front Pharmacol. 2021;12:680512. doi:10.3389/fphar.2021.680512. 34122107 PMC8193053

[14] Sun W, Li A, Wang Z, Sun X, Dong M, Qi F, Wang L, Zhang Y, Du P. Tetramethylpyrazine alleviates acute kidney injury by inhibiting NLRP3/HIF‑1α and apoptosis. Mol Med Rep. 2020;22:2655–2664. doi:10.3892/mmr.2020.11378. 32945382 PMC7453617

[15] Li S, Xu Y, He S, Li X, Shi J, Zhang B, Zhu Y, Li X, Wang Y, Liu C, et al. Tetramethylpyrazine ameliorates endotoxin-induced acute lung injury by relieving golgi stress via the Nrf2/HO-1 signaling pathway. BMC Pulm Med. 2023;23(1):286. doi:10.1186/s12890-023-02585-3. 37550659 PMC10408181

[16] Zhang Q, Liu J, Duan H, Li R, Peng W, Wu C. Activation of Nrf2/HO-1 signaling: an important molecular mechanism of herbal medicine in the treatment of atherosclerosis via the protection of vascular endothelial cells from oxidative stress. J Adv Res. 2021;34:43–63. doi:10.1016/j.jare.2021.06.023. 35024180 PMC8655139

[17] Siracusa R, D’Amico R, Cordaro M, Peritore AF, Genovese T, Gugliandolo E, Crupi R, Impellizzeri D, Cuzzocrea S, Fusco R, et al. The methyl ester of 2-cyano-3,12-dioxooleana-1,9-dien-28-oic acid reduces endometrial lesions development by modulating the NFkB and Nrf2 pathways. Int J Mol Sci. 2021;22(8):22. doi:10.3390/ijms22083991. PMC8069675 33924360

[18] Su W, Cui H, Wu D, Yu J, Ma L, Zhang X, Huang Y, Ma C. Suppression of TLR4-MyD88 signaling pathway attenuated chronic mechanical pain in a rat model of endometriosis. J Neuroinflammation. 2021;18(1):65. doi:10.1186/s12974-020-02066-y. 33673857 PMC7934423

[19] Li Y, Meng X, Fu X, An M, Liu H, Ma Y, Li Q, Hao G, Ma Y, Zhang Y, et al. Bushen wenyang huayu decoction targets TLR4/NF-κB mediated autophagy to treat endometriosis effectively. Evid Based Complement Alternat Med. 2022;2022:1–11. doi:10.1155/2022/4263417. PMC9699773 36437825

[20] Talebi H, Farahpour MR, Hamishehkar H. The effectiveness of Rutin for prevention of surgical induced endometriosis development in a rat model. Sci Rep. 2021;11(1):7180. doi:10.1038/s41598-021-86586-4. 33785814 PMC8010059

[21] Zong C, Sun L, Xu X, Xue X, Emran TB. Huayu sanjie enema liquid relieves pain in endometriosis model rats by inhibiting inflammation, peripheral sensitization, and pelvic adhesion. Evid Based Complement Alternat Med. 2022;2022:1–10. doi:10.1155/2022/5256578. PMC9256397 35800014

[22] Zheng W, Li M, Wang Y, Lv B, Zhang X, Chen L, Zhu K, Wang Z, Li B, Xiao W, et al. Guizhi fuling capsule exhibits antidysmenorrhea activity by inhibition of cyclooxygenase activity. Evid Based Complement Alternat Med. 2020;2020(1):8607931. doi:10.1155/2020/8607931. 32595743 PMC7262657

[23] Liu J, Yang D, Piao C, Wang X, Sun X, Li Y, Zhang S, Wu X. UPLC-Q-TOF/MS based plasma metabolomics for identification of paeonol’s metabolic target in endometriosis. Molecules. 2023;28(2):28. doi:10.3390/molecules28020653. PMC9864815 36677710

[24] Scutiero G, Iannone P, Bernardi G, Bonaccorsi G, Spadaro S, Volta CA, Greco P, Nappi L. Oxidative stress and endometriosis: a systematic review of the literature. Oxid Med Cell Longev. 2017;2017(1):7265238. doi:10.1155/2017/7265238. 29057034 PMC5625949

[25] Gegotek A, Skrzydlewska E. Ascorbic acid as antioxidant. Vitam Horm. 2023;121:247–270. 36707136 10.1016/bs.vh.2022.10.008

[26] Li QF, Zhu YS, Jiang H, Xu H, Sun Y. Heme oxygenase-1 mediates the anti-inflammatory effect of isoflurane preconditioning in lps-stimulated macrophages. Acta Pharmacol Sin. 2009;30(2):228–234. doi:10.1038/aps.2008.19. 19122672 PMC4002462

[27] Wei X, Zhang T, Ma C, Zhang M, Jin L, Ma X, Zhang Z. TRIM27 ameliorates ischemic stroke by regulating NLRP3 inflammasome-mediated pyroptosis via the Akt/Nrf2/HO-1 signaling. Exp Neurol. 2024;371:114599. doi:10.1016/j.expneurol.2023.114599. 37914066

[28] Lamceva J, Uljanovs R, Strumfa I. The main theories on the pathogenesis of endometriosis. Int J Mol Sci. 2023;24(5):24. doi:10.3390/ijms24054254. PMC10001466 36901685

[29] Salliss ME, Farland LV, Mahnert ND, Herbst-Kralovetz MM. The role of gut and genital microbiota and the estrobolome in endometriosis, infertility and chronic pelvic pain. Hum Reprod Update. 2021;28(1):92–131. doi:10.1093/humupd/dmab035. 34718567

[30] Zhang C, Zhang Y, Pan H, Tan Y, Wei Q, Dai X, Wei J, Chen Y. Combination of ferulic acid, ligustrazine and tetrahydropalmatine attenuates epithelial-mesenchymal transformation via Wnt/β-catenin pathway in endometriosis. Int J Biol Sci. 2021;17(10):2449–2460. doi:10.7150/ijbs.60167. 34326686 PMC8315018

[31] Clower L, Fleshman T, Geldenhuys WJ, Santanam N. Targeting oxidative stress involved in endometriosis and its pain. Biomolecules. 2022;12(8):12. doi:10.3390/biom12081055. PMC9405905 36008949

[32] Ali SS, Ahsan H, Zia MK, Siddiqui T, Khan FH. Understanding oxidants and antioxidants: classical team with new players. J Food Biochem. 2020;44(3):e13145. doi:10.1111/jfbc.13145. 31960481

[33] Li Y, Zhu Z, Zhang T, Zhou Y. Ligustrazine attenuates inflammation and oxidative stress in a rat model of arthritis via the Sirt1/NF-κB and Nrf-2/HO-1 pathways. Arch Pharm Res. 2019;42(9):824–831. doi:10.1007/s12272-018-1089-0. 30448958

[34] Li Z, Shan L, Yu P. Preventive effect of tetramethylpyrazine on nitroglycerin-tolerance in rats by improving oxidative stress and ribosome homeostasis. Biochem Biophys Res Commun. 2022;618:141–147. doi:10.1016/j.bbrc.2022.06.013. 35724458

[35] Dhaliwal J, Dhaliwal N, Akhtar A, Kuhad A, Chopra K. Tetramethylpyrazine attenuates cognitive impairment via suppressing oxidative stress, neuroinflammation, and apoptosis in type 2 diabetic rats. Neurochem Res. 2022;47(8):2431–2444. doi:10.1007/s11064-022-03640-x. 35665448

[36] Hussain T, Tan B, Yin Y, Blachier F, Tossou MC, Rahu N. Oxidative stress and inflammation: what polyphenols can do for us? Oxid Med Cell Longev. 2016;2016(1):7432797. doi:10.1155/2016/7432797. 27738491 PMC5055983

[37] Hung SW, Zhang R, Tan Z, Chung JPW, Zhang T, Wang CC. Pharmaceuticals targeting signaling pathways of endometriosis as potential new medical treatment: a review. Med Res Rev. 2021;41(4):2489–2564. doi:10.1002/med.21802. 33948974 PMC8252000

[38] Monsanto SP, Edwards AK, Zhou J, Nagarkatti P, Nagarkatti M, Young SL, Lessey BA, Tayade C. Surgical removal of endometriotic lesions alters local and systemic proinflammatory cytokines in endometriosis patients. Fertil Steril. 2016;105(4):968–977 e5. doi:10.1016/j.fertnstert.2015.11.047. 26698677 PMC4851862

[39] Coll RC, Schroder K, Pelegrin P. NLRP3 and pyroptosis blockers for treating inflammatory diseases. Trends Pharmacol Sci. 2022;43(8):653–668. doi:10.1016/j.tips.2022.04.003. 35513901

[40] Swanson KV, Deng M, Ting JP. The NLRP3 inflammasome: molecular activation and regulation to therapeutics. Nat Rev Immunol. 2019;19(8):477–489. doi:10.1038/s41577-019-0165-0. 31036962 PMC7807242

[41] Zeng X, Liu D, Huo X, Wu Y, Liu C, Sun Q. Pyroptosis in NLRP3 inflammasome-related atherosclerosis. Cell Stress. 2022;6(10):79–88. doi:10.15698/cst2022.10.272. 36304814 PMC9549904

[42] Song N, Li T. Regulation of NLRP3 inflammasome by phosphorylation. Front Immunol. 2018;9:2305. doi:10.3389/fimmu.2018.02305. 30349539 PMC6186804

[43] Zhang M, Shi Z, Peng X, Cai D, Peng R, Lin Y, Dai L, Li J, Chen Y, Xiao J, et al. NLRP3 inflammasome-mediated pyroptosis induce notch signal activation in endometriosis angiogenesis. Mol Cell Endocrinol. 2023;574:111952. doi:10.1016/j.mce.2023.111952. 37268099

[44] Ma X, Ruan Q, Ji X, Yang J, Peng H. Ligustrazine alleviates cyclophosphamide-induced hepatotoxicity via the inhibition of Txnip/Trx/NF-κB pathway. Life Sci. 2021;274:119331. doi:10.1016/j.lfs.2021.119331. 33716060

[45] Chang CY, Kao TK, Chen WY, Ou YC, Li JR, Liao SL, Raung S-L, Chen C-J. Tetramethylpyrazine inhibits neutrophil activation following permanent cerebral ischemia in rats. Biochem Biophys Res Commun. 2015;463(3):421–427. doi:10.1016/j.bbrc.2015.05.088. 26043690

[46] Zhang L, Wang X, Lu X, Ma Y, Xin X, Xu X, Wang S, Hou Y. Tetramethylpyrazine enhanced the therapeutic effects of human umbilical cord mesenchymal stem cells in experimental autoimmune encephalomyelitis mice through Nrf2/HO-1 signaling pathway. STEM Cell Res Ther. 2020;11(1):186. doi:10.1186/s13287-020-01700-z. 32430010 PMC7238657

[47] Li B, Nasser MI, Masood M, Adlat S, Huang Y, Yang B, Luo C, Jiang N. Efficiency of traditional Chinese medicine targeting the Nrf2/HO-1 signaling pathway. Biomed Pharmacother. 2020;126:110074. doi:10.1016/j.biopha.2020.110074. 32163746

[48] Huang C, Zhang C, Yang P, Chao R, Yue Z, Li C, Guo J, Li M. Eldecalcitol inhibits LPS-Induced NLRP3 inflammasome-dependent pyroptosis in human gingival fibroblasts by activating the Nrf2/HO-1 signaling pathway. Drug Des Devel Ther. 2020;14:4901–4913. doi:10.2147/DDDT.S269223. PMC7671541 33223823

[49] Kang JY, Xu MM, Sun Y, Ding ZX, Wei YY, Zhang DW, Wang Y-G, Shen J-L, Wu H-M, Fei G-H, et al. Melatonin attenuates lps-induced pyroptosis in acute lung injury by inhibiting NLRP3-GSDMD pathway via activating Nrf2/HO-1 signaling axis. Int Immunopharmacol. 2022;109:108782. doi:10.1016/j.intimp.2022.108782. 35468366

[50] Qiao J, Ma H, Chen M, Bai J. Vitamin D alleviates neuronal injury in cerebral ischemia-reperfusion via enhancing the Nrf2/HO-1 antioxidant pathway to counteract NLRP3-mediated pyroptosis. J Neuropathol Exp Neurol. 2023;82(8):722–733. doi:10.1093/jnen/nlad047. 37403613

[51] D’Amico R, Impellizzeri D, Cordaro M, Siracusa R, Interdonato L, Crupi R, Gugliandolo E, Macrì F, Di Paola D, Peritore AF, et al. Regulation of apoptosis and oxidative stress by oral boswellia serrata gum resin extract in a rat model of endometriosis. Int J Mol Sci. 2022;23(23):23. doi:10.3390/ijms232315348. PMC9736785 36499679

